# Bendamustine and Rituximab, as First Line Treatment, in Intermediate, High Risk Splenic Marginal Zone Lymphomas of Elderly Patients

**DOI:** 10.4084/MJHID.2016.030

**Published:** 2016-07-01

**Authors:** Roberto Castelli, Antonio Gidaro, Giorgio Lambertenghi Deliliers

**Affiliations:** 1 Department of Biomedical and Clinical Sciences Luigi Sacco, University of Milan, Luigi Sacco Hospital Milan, Milan, Italy; 2 Fondazione Mattarelli Milano

## Abstract

**Background:**

Splenic marginal zone lymphoma (SMZL) is a chronic B-cell lymphoproliferative disorder, comprising less than 2% of non-Hodgkin’s lymphomas, and affecting mainly middle-aged and elderly patients with a median survival of >10 years. The typical clinical features of SMZL include splenomegaly. Treatment should be patient-tailored and can range from a ‘watchful waiting’ approach for asymptomatic patients without cytopenias to surgery, localized radiation therapy or immuno/chemotherapies. Recently, the combination of rituximab and Bendamustine (R-Benda) has been defined as highly active in patients with follicular lymphomas, but little is known about the efficacy of R-Benda in SMZL.

**Aim of the study:**

The purpose of this retrospective study was to report our experience on the efficacy of R-Benda as first line treatment in 23 consecutive elderly SMZL patients.

**Results:**

All patients had a complete resolution of splenomegaly along with restoration of their blood counts. Nineteen patients (83%) achieved a complete response (CR) to therapy; three patients (13%) achieved a partial response (PR).Ten patients (43%) obtained molecular remission. Toxicities were mild and mainly haematological and result in dose reductions for fourteen patients.

**Conclusions:**

Our data suggest a high activity and good tolerance of R-Benda, despite dose reduction due to potential toxicity.

## Introduction

Splenic Marginal Zone Lymphoma (SMZL) is a low-grade B-cell lymphoma considered as a rare lymphoproliferative disease (2% of all non-Hodgkin’s lymphomas). It is estimated that it represents for most cases of otherwise unclassifiable chronic lymphoid B-cell CD 5-lymphoprolipherative disorders.^1^ SMZL is characterized by an almost exclusive involvement of the spleen and bone marrow without lymphadenopathy. SMZL has a relatively indolent course with a median survival of >10 years,^2^–^3^ but some patients can suffer a more rapid course. In fact about 25% of patients pursues an aggressive course and most of them die of lymphoma progression within 3–4 years.^4^

Lymphonode and extra nodal involvement; high lymphocyte count; anemia; and thrombocytopenia on the diagnosis or when they are developed during the course of the disease are considered prognostic criteria of progression.^5^–^6^ The Integruppo Italiano Linfomi (IIL) have proposed clinical score based on three parameters: anaemia, elevated LDH values, and hypoalbuminemia to evaluate the risk of progression and found that patients are showing two or more of the above adverse prognostic factors have a median life expectancy of fewer than five years.^7^ The patients are grouped into three categories of increasing severity: (1) low risk when none of the risk factors are present (5-year lymphoma specific survival of 88%); (2) intermediate risk when one risk factor is present (5-year lymphoma specific survival of 73%); and (3) high risk when two or more risk factors are present (5-year lymphoma specific survival of 50%). The patients enrolled in this study all belonged to intermediate or high risk score. Treatment should be patient tailored and can range from a ‘watchful waiting’ approach for asymptomatic patients without cytopenias to splenectomy or localized radiation therapy. Recently, the combination of rituximab and Bendamustine (R-Benda) has been defined as highly active in patients with indolent lymphomas,^8^ but little is known about the efficacy of R-Benda in SMZL, especially in elderly patients due to the rarity of the disease.

## Patients and Methods

Twenty-three patients with splenic marginal zone B cells were included in this retrospective study approved by Ethical Board of our University Hospital. The diagnosis was obtained by a combination of bone marrow histology, clinical findings, and immunophenotype. Lymphoma cells were expressing positivity for CD19+, CD20+, CD79a+, CD5−, CD10−, CD23−, BCL6−, cyclin D1-.None of the patients underwent splenectomy. Staging procedures were performed as routinely at the time of diagnosis and before treatment with rituximab/bendamustine. Polymerase chain reaction (PCR) analysis of blood and/or bone marrow mononuclear cells for the presence of IgH, rearrangement was also performed prior to treatment initiation and at the time of remission.^9^ Clinical data were collected: patient’s demographics complete blood cell count, albumin, plasma lactic dehydrogenase (LDH), and hepatitis B and C serology, bone marrow findings. B symptoms, performance status (according to Eastern Cooperative Oncology Group performance status, ECOG), date of diagnosis, treatment, treatment response, date of relapse, date of the last follow-up and cause of death were also collected. Prognostication was performed according to the suggestion of IIL.^7^ The schedule of R–B was the following: six courses of Bendamustine at 90 mg/m^2^ or 70 mg/m^2^ (depending on clinician’s choice) on days 1 and 2 of each 28-day cycle and rituximab 375 mg/m^2^ on day 1 of each cycle. Post-treatment evaluation was performed at 8 weeks after the completion of the sixth rituximab/Bendamustine infusion.

Response criteria were as follows: Complete remission (CR): Complete resolution of symptoms, normalization of peripheral blood counts, the absence of detectable disease by clinical staging including bone marrow biopsy, and no evidence of clonal B-cell population by blood and bone marrow immunophenotype. Molecular remission: Absence of IgH rearrangement in bone marrow samples in previously positive patients. Complete remission unconfirmed (CRu): Complete resolution of symptoms and absence of detectable disease by clinical staging in patients who did not undergo bone marrow evaluation post-rituximab/Bendamustine. Partial remission (PR): At least 50% decrease in the spleen size and the percentage of bone marrow infiltration (evaluated by trephine biopsy) along with the improvement of blood counts over baseline. Failure: Any lesser response than described above.

## Results and discussion

A total of 23 patients (15 females and 8 males) with SMZL median age 75 years (range 65–88 years) were enrolled. Clinical and laboratory parameters are presented in table 1. Forty teen patients (61%) had haemoglobin levels below 11 g/dl, thrombocytopenia in nine patients (39%), all patients had bone marrow lymphatic involvement, neutropenia was present in eight patients (35%) of patients.

Monoclonal serum immunoglobulin was present 13 patients (57%), and the most represented Ig Isotype was IgM. Autoimmune manifestations or concomitant autoimmune disease were present in nine patients (39%). Splenectomy was performed in none of our patients. In all patients, Rituximab-Bendamustine was given as first line treatment in a median time to diagnosis of 60 days (range 1–75) after diagnosis of SMZL. Twenty-two patients underwent 6 cycles of Bendamustine and Rituximab therapy; one patient has not completed the 6 cycle of treatment due to toxicities. Twelve patients (52%) of our study population experienced a Bendamustine dose reduction to 70 mg/m^2^ for two days. Responses were assessed after 3 cycles and after 6 cycles of chemotherapy. Bone marrow biopsy performed after chemotherapy is available for twenty-two patients. Nineteen patients (83%) achieved a complete response (CR) to therapy; three patients (13%) achieved a partial response (PR). One patient (4.3%) had disease progression 18 months after initial partial remission and was shift to R CHOP (Rituximab, Cyclofosfamide, Doxorubicine, Vincristine, and Prednisone) obtaining complete remission. Among patients with CR, six patients (26%) had a partial response after 3 cycles and obtained CR after 3 additional cycles of therapy. Ten patients (43%) obtained molecular remission. (Absence of IgH rearrangement in bone marrow samples in previously positive patients). The median time to clinical response in terms resolutions of clinical symptoms was 2 weeks (range 1–6) and the median time to haematological response was 4 weeks (range 2–6 weeks). Table 2 show changes in clinical and haematological parameters in 23 patients with splenic marginal zone lymphomas, prior and after treatment with Bendamustine and Rituximab. After treatment, we obtained resolution of autoimmune haemolytic anaemia and acquired deficiency of C1-INH. Patients were followed for a median time of 24 months (range 12–50 months). The treatment was generally well tolerated. One patient experienced infusion related side effects, including chills, rigors, bronchospasm and back pain treated with discontinuation of rituximab and reinfusion at a lower rate. Patients experienced hematologic toxicities: neutropenia in 16 patients (70%), one patient developed mild to moderate lasting neutropenia for 2 months, eight patients (35%) experienced thrombocytopenia. The most frequent non-haematological adverse events of any grade included nausea in 15 patients (65%), infection in 16 patients (70%). Some of the infections included three episodes of CMV infection and three episodes of herpes zoster. Eight patients (35%) presented vomiting; pyrexia was observed in eight patients (35%). Seven patients (30%) experienced grade 3 neutropenia and infection in 16%. Slight skin reactions responding to antihistaminics were seen in 3 patients (13%) during the first cycles of Bendamustine administration and disappeared thereafter.

MZL is a rare group of NHL, which is indolent in nature with subgroups, SMZL, NMZL, and MALT lymphoma. These lymphomas share some similarities but are different in their biological nature and hence behave differently depending on their location. SMZL affects the elderly with a median age of 70 years, the median survival exceeds ten years, and most patients can be effectively managed for many years with a watchful waiting policy. Splenectomy has been traditionally considered the standard of care for the treatment of patients with SMZL.^10^

However, being a major surgical procedure, it carries a high risk of complications. For patient or those who show a spread of the disease to lymphonode or other extranodal sites or otheradverse prognostic factors, a systemic treatment may be appropriate. The introduction of anti-CD20 antibody rituximab significantly improved the treatment outcomes of SMZL due to the strong expression of CD 20 antigen in SMZL cells. Kalpadakis and colleagues^11^ showed that the use of rituximab as a single agent yielded an overall response rate (ORR) of 100%, with 69% complete response (CR), 19% unconfirmed response and 12% partial response (PR). In about 25% of cases, the disease pursues an aggressive course, and most patients die of lymphoma progression within 3–4 years. Recently the IIL have proposed a clinical score to evaluate the risk of lymphoma progression. The challenge of our study was to evaluate if Rituximab Bendamustine treatment, as first line chemotherapy, may be appropriate in high intermediate risk elderly patients with SMZL.

Nineteen patients (83%) achieved a complete response (CR), three patients (13%) achieved a partial response (PR). One patient (4.3%) had disease progression, 18 months after initial partial remission and was shift to R CHOP (Rituximab, Cyclofosfamide, Doxorubicine, Vincristine and Prednisone) obtaining complete remission. Ten patients (43%) obtained molecular remission. Our study suggests that R-Benda is effective and tolerable (despite the need for dose reduction in elderlies) in patients with SMZL.

The present study has some limitations (retrospective, and follow be too short to asses progression free survival and without statistical power to asses superiority of R Benda as compared to Rituximab alone), and if these results will be confirmed by larger and prospective studies it should be of benefits in SMZL patients carrying risk factor for an aggressive course.

## Figures and Tables

**Table 1:** Clinical characteristics of splenic marginal zone lymphomas patients treated with Rituximab and Bendamustine.

	Sex/Age	Risk class	Autoimmune disease	Performance status	Paraproteinemia	N of cycles	Cycle 3	Cycle 6	Overall response	Follow up months
1	F/71	II	Acquired C1-INH deficiency	0	IgM K	6	CR	CR	CR	24
2	F/72	II	Acquired C1-INH deficiency	0	IgM k	6	CR	CR	CR	30
3	M/68	III	none	1	IgM k	3	PR	-	PR	12
4	M/71	III	none	1	IgG K	6	PR	CR	CR	19
5	M/88	III	Rheumatoid arthritis	0	IgM K	6	CR	CR	CR	20
6	F/88	II	None	0	IgM K	6	PR	CR	CR	24
7	F/78	II	Lupus anticoagulant	0	None	6	PR	CR	CR	28
8	F/82	III	Acquired C1-INH deficiency	0	IgM k	6	PR	CR	CR	40
9	F/80	II	none	0	none	6	PD	PD	PD	24 relapsed 18 months after initial PR
10	F/65	II	Sjogren’sdiseas	2	none	6	CR	CR	CR	15
11	M/68	III	Hashimoto disease	0	none	4	CR	CR	CR	20
12	M/78	II	None	1	ND	6	CR	CR	CR	24
13	M/74	III	none	1	none	6	CR	CR	CR	20
14	F/80	III	none	0	IgG Kappa	6	PR	CR	CR	32
15	F/66	II	Haemolytic anaemia	0	IgM kappa	6	PR	PR	PR	30
16	F/88	III	none	0	none	6	PR	CR	CR	32
17	M/65	III	none	0	IgM Kappa	6	PR	CR	CR	24
18	M/74	III	none	1	none	6	CR	CR	CR	20
19	F/80	III	none	0	IgG Kappa	6	PR	CR	CR	32
20	F/65	II	Sjogren’s disease	1	IgM k	6	CR	CR	CR	26
21	F/70	II	None	1	None	6	PR	CR	CR	12
22	F/65	II	None	1	None	6	PR	CR	CR	23
23	F/88	II	none	1	IgM K	6	PR	PR	PR	26

No number, PS performance status, ORR overall response CR complete remission, PR partial remission, PD progressive disease, ND no data

**Table 2:** Changes in clinical and haematological parameters in 23 patients with splenic marginal zone lymphomas, prior and after treatment with Bendamustine and Rituximab.

	Before the treatment with Bendamustine and Rituximab	At the end of the treatment with Bendamustine and Rituximab
Haemoglobin (g/dl)	10.5 (7.4–14.5)	12.5 ( 10.6–14.7)
Platelet count (× 10 9/L)	110 (47–350)	160 (120–470)
Lymphocyte count (× 10 9/L)	5 (0.70–23)	1.6 (0.8–3.0)
Neutrophils (× 10 9/L)	2.5 (1.3–5)	4.0 (1.5–6.0)
Bone marrow infiltration (% of lymphoid cells)	44 (15–70)	0 (0–5)

Spleen size (median cm (range)	18 (3–20)	10 (3–10)
